# Dynamic TF-lncRNA Regulatory Networks Revealed Prognostic Signatures in the Development of Ovarian Cancer

**DOI:** 10.3389/fbioe.2020.00460

**Published:** 2020-05-13

**Authors:** Qiuyan Guo, Junwei Wang, Yue Gao, Xin Li, Yangyang Hao, Shangwei Ning, Peng Wang

**Affiliations:** ^1^College of Bioinformatics Science and Technology, Harbin Medical University, Harbin, China; ^2^Department of Gynecology, The First Affiliated Hospital, Harbin Medical University, Harbin, China; ^3^Department of Respiratory Medicine, The Second Affiliated Hospital, Harbin Medical University, Harbin, China

**Keywords:** ovarian cancer, lncRNAs, transcription factors, prognostic signature, functional analysis

## Abstract

The pathological development of ovarian cancer (OC) is a complex progression that depends on multiple alterations of coding and non-coding genes. Therefore, it is important to capture the transcriptional-regulating events during the progression of OC development and to identify reliable markers for predicting clinical outcomes in patients. A dataset of 399 ovarian serous cystadenocarcinoma patients at different stages from The Cancer Genome Atlas (TCGA) was analyzed. Stage-specific transcription factor (TF)-long non-coding RNA (lncRNA) regulatory networks were constructed by integrating high-throughput RNA molecular profiles and TF binding information. Systematic analysis was performed to characterize the TF-lncRNA-regulating behaviors across different stages of OC. Cox regression analysis and Kaplan-Meier survival curves were used to evaluate the prognostic efficiency of TF-lncRNA regulations and cliques. The stage-specific TF-lncRNA regulatory networks at three OC stages (II, III, and IV) exhibited common structures and specific topologies of risk TFs and lncRNAs. A TF-lncRNA activity profile across different stages revealed that TFs were highly stage-selective in regulating lncRNAs. Functional analysis indicated that groups of TF-lncRNA interactions were involved in specific pathological processes in the development of OC. In a STAT3-FOS co-regulating clique, the TFs STAT3 and FOS were selectively regulating target lncRNAs across different OC stages. Further survival analysis indicated that this TF-lncRNA biclique may have the potential for predicting OC prognosis. This study revealed the topological and dynamic principles of TF-lncRNA regulatory networks and provided a resource for further analysis of stage-specific regulating mechanisms of OC.

## Introduction

Ovarian cancer (OC) is the most common disease worldwide with the highest death rate of all gynecological tumors (Siegel et al., 2018). Due to its asymptomatic stages and rapid metastasis to the peritoneum, most patients have already developed metastases by the time they are first diagnosed (Bowtell, 2010). Despite advances in chemotherapy and surgical treatment, the prognosis for OC patients remains unsatisfactory, and only 30% of patients survive as long as 5 years after initial diagnosis (Rustin et al., 2011). The pathological development of OC is a complex progression that depends on multiple alterations of oncogenes and tumor suppressors. Although our knowledge of OC is increasingly expanding, the precise molecular mechanisms underlying this complex disease are still not fully understood. Therefore, it is important to understand mechanisms promoting the progression of OC and to identify reliable markers for predicting clinical outcomes in patients.

In recent years, emerging evidences have shown the importance of long non-coding RNAs (lncRNAs) as new regulators of many physical or pathological processes (Gibb et al., 2011). Accumulating evidence suggests that variations in lncRNA expression play critical roles in the progression of OC tumors. Transcription factors (TFs) perform key functions in controlling lncRNA expression through sequence-specific binding sites in lncRNA transcripts. The differential expression of TFs and their downstream targets have been proved to be associated with the progression of multiple types of cancers. For example, the TF Oct4 promotes development of osteosarcoma by regulating lncRNA AK055347 (Fan et al., 2017). Some oncogenes and tumor suppressors such as TP53, STAT3, and JUN were found to be associated with glioma development (Wei et al., 2015). In a previous study, four differentially expressed TFs (MYC, FOXO1, FOXM1, and SMAD) were found to regulate 16 lncRNAs involved in the pathogenesis of obliterative bronchiolitis (Dong et al., 2015). With the acquisition of more genome-wide annotations, large-scale analysis based on the bipartite network has been performed to dissect regulatory mechanisms between coding and non-coding genes (Liu et al., 2019; Zhao et al., 2019). Previous studies have found that human TF-regulating networks are highly specific to different types of cells (Neph et al., 2012) and appear to undergo dynamic reconfiguration in specific contexts (Carro et al., 2010). By constructing a lncRNA-mediated feed-forward loop network, Ning et al. demonstrated that some TF-lncRNA interactions were involved in prognostic motifs in different cancers (Ning et al., 2016). These studies have significantly enhanced our understanding of the TF-regulating mechanisms underlying disease progression. Unfortunately, there has only been limited work on the dynamic TF-lncRNA networks involved in OC development.

To address this issue, we analyzed a dataset of 399 ovarian serous cystadenocarcinoma patients at different stages from The Cancer Genome Atlas (TCGA) (Cancer Genome Atlas Research Network, 2011). By integrating high-throughput RNA molecular profiles and TF binding information, we constructed TF-lncRNA regulatory networks and performed systematic analysis for each stage of OC patients. We found that three stage-specific TF-lncRNA regulatory networks exhibited common structures and specific topologies of risk TFs and lncRNAs. A TF-lncRNA activity profile across different stages revealed that TFs were highly stage-selective in regulating lncRNAs. Functional analysis indicated that groups of TF-lncRNA interactions tend to be involved in specific pathological processes in the development of OC, such as uncontrolled tumor cell growth at stage II, immune cell activation and differentiation processes at stage III and chondroitin sulfate biosynthesis at stage IV. Based on network clique analysis, we found that TF-lncRNA bicliques were associated with several cancer hallmarks. In a STAT3-FOS co-regulating network clique, the TFs STAT3 and FOS selectively regulated target lncRNAs in different OC stages. Survival analysis indicated that the TF-lncRNA biclique may have a potential role for predicting OC prognosis. The prognostic efficiency was further evaluated in the independent cohorts GSE26193 and GSE9891. In summary, our systematic analysis not only sheds new light on dynamic regulatory mechanisms of TF-lncRNA interactions, but may also help in OC prognosis stratification and discovery of therapeutic targets.

## Materials and Methods

### The Expression Datasets of mRNAs and lncRNAs in OC

The expression profile of whole-genome transcripts, including coding and non-coding RNAs, was derived from a previous study (Akrami et al., 2013), which calculated RPKM values using raw RNA-sequencing libraries from the TCGA OC dataset. Transcript annotations of coding mRNAs and lncRNAs were downloaded from GENCODE (v19) (Harrow et al., 2012). Finally, 29,250 mRNAs and 10,412 lncRNAs were identified from RNA-sequencing data. Another two independent OC datasets (GSE26193 and GSE9891) were downloaded from publicly available Gene Expression Omnibus (GEO) database, including 107 and 278 patients, respectively. Both of the microarray datasets were tested using the Affymetrix Human Genome U133 Plus 2.0 Array platform. Patients with well-annotated clinical follow-up information were retained for further analysis. To derive lncRNAs and mRNAs expression from microarray datasets, we mapped probe sequences to the human genome of GENCODE (v19) by using SeqMap software (Jiang and Wong, 2008). Probes that were uniquely mapped to lncRNA transcripts were reserved. No mismatches were allowed in the mapping procedure. If multiple probes were mapped to the same gene, the mean expression value was calculated. This procedure has been performed in previous studies to obtain lncRNA expression from microarray datasets (Du et al., 2013). Finally, 16,345 mRNAs and 3,308 lncRNAs were identified from microarray data.

### Clinical Characteristics of OC Patients

The clinical and pathological characteristics of OC patients were downloaded from the TCGA data portal. The staging and grading information was collected based on criteria of the International Federation of Gynecologists and Obstetricians and the World Health Organization. A subset of 399 patients (including 20 stage-II, 318 stage-III and 61 stage-IV) with clinical follow-up information were retained for survival analysis (Table 1).

**Table 1:** Clinicopathologic characteristics of ovarian cancer patients (*n* = 399).

**Characteristics**	**Number of patients**			***P***
	**All patients *n* = 399**	**Training set *n* = 200**	**Testing set *n* = 199**	
Stage				0.13^a^
II	20	6	14	
III	318	160	158	
IV	61	34	27	
Age				0.95^b^
Mean ± SD	59.54 ± 11.35	59.50 ± 10.91	59.58 ± 11.81	
Range	30–87	34–84	30–87	
Histological grade				0.66^a^
GX	7	3	4	
G1	1	1	0	
G2	46	20	26	
G3	344	175	169	
G4	1	1	0	
Residual tumor diameter (cm)				0.87^a^
<1	192	98	94	
≥1	101	51	50	
Unknown	106	51	55	
Lymph node metastasis				0.27^a^
Absent	54	22	32	
Present	102	50	52	
Unknown	243	128	115	
Survival (month)				0.93^b^
Mean ± SD	34.35 ± 27.70	34.22 ± 28.33	34.47 ± 27.11	
Range	0.30–182.70	0.77–182.70	0.30–131.77	
State				0.29^a^
Living		81	92	
Death		119	107	

a *P-values were determined using Chi-square test or Fisher's exact test when appropriate*.

b *P-values were determined using Student's t-test*.

### Identification of TF-lncRNA Regulatory Interactions

To identify potential TF-lncRNA relationships, we used an integrated pipeline, which was developed and utilized in our previous studies (Ning et al., 2014, 2016). The locations and conserve scores based on multiz46way alignments of transcription factor binding sites were downloaded from the UCSC table browser (Karolchik et al., 2003). Genome locations of conserved transcription factor binding sites were mapped to the promoter region (5 kb upstream to 1 kb downstream of the transcription start site) for each lncRNA. A total of 690 ChIP-Seq datasets in different cell lines and tissues were downloaded from the ENCODE project (Wellcome Trust Case Control Consortium, 2007). The peaks of sequenced reads were computed using the PeakSeq method (Rozowsky et al., 2009), which identifies enriched peaks by comparing each ChIP-Seq dataset to the corresponding control experiments. The peaks located in the promoter region were retained. By integration of TF-lncRNA relationships from conserved transcription factor binding sites and ChIP-Seq datasets, 58,119 potential TF-lncRNA interactions among 151 TFs and 3,981 lncRNAs were obtained. To identify the actual TF-lncRNA regulatory relationships, we performed co-expression analysis in different OC stages. Pearson correlation coefficients were calculated for each of the potential TF-lncRNA pairs based on their expression profiles in patients at stages II, III, and IV. We used Pearson coefficient >0 and false discovery rate (FDR) <0.05 as thresholds to capture positive relationship between TFs and lncRNAs. Specifically, 2,852 edges between 144 TFs and 1,261 lncRNAs (Table S1), 7,395 edges between 147 TFs and 1,797 lncRNAs (Table S2) and 3,908 edges between 145 TFs and 1,403 lncRNAs (Table S3) were constructed for patients in stages II, III, and IV, respectively.

### Network Illustration and Topological Analysis

Cytoscape software (v3.1.1) was used to construct and illustrate the TF-lncRNA regulatory networks. Several topological properties, such as the node degree, topological coefficient and betweenness centrality, were analyzed by the built-in Network Analyzer tool in Cytoscape. The degree for a node is the number of its network neighbors. The topological coefficient for node n is calculated as an average of J(n,m)/e, where m is the one-step-neighbor of n, J(n,m) is the number of nodes shared by m and n, and e is the number of neighbors of n. Betweenness centrality indicates the network centrality of a node and is equal to the proportion of shortest paths from all nodes to all others that pass through this node. The biclique module is a complete bipartite graph in which all TFs are connected with all lncRNAs. The TF-lncRNA-regulating bicliques were identified using an R package for enumerating maximal complete bipartite graphs (Zhang et al., 2014).

### The Activity Scores of TF-lncRNA Interactions

For a TF-lncRNA interaction, the co-expression coefficient in each stage of OC was used as the activity score and normalized by the *z*-score method:

(1) $$\begin{array}{r} \text{z}=\frac{x-\mu }{\sigma } \end{array}$$

Where x is the activity score for a certain TF-lncRNA pair, μ is the mean value of all activity scores in a stage and σ is the standard deviation. In each stage, the *z*-scores were further transformed by subtracting the minimum value. After normalization, the activity scores in each stage were required to have similar distributions (Table S4 and Figure S1).

### The Specificity Scores of TF-lncRNA Interactions and Cliques

The specificity of each TF-lncRNA interaction across different stages of OC was determined by the specificity score as:

(2) $$\begin{array}{r} Specificity\;score=\frac{\sum_{i=1}^{N}\left(1-x_{i}\right)}{N-1} \end{array}$$

Where N is the number of OC stages and x_i_ is a component normalized to the maximum value of the Pearson correlation coefficient. For a TF-lncRNA regulatory clique, the specificity is evaluated by the average value of specificity scores for all TF-lncRNA relationships in the clique. According to a previous study (Wang et al., 2015), the range of specificity score is from 0 to 1, indicating low to high specificity. The specificity scores of TF-lncRNA interactions are listed in Table S5.

### Construction of the Risk Score Model

To evaluate the efficiency of TF-lncRNA cliques in predicting the survival of OC patients, the 399 OC patients were randomly divided into two groups that were used as training (*n* = 200) and testing (*n* = 199) datasets (Table S6). There were no significant differences in the clinical characteristics between two groups of patients (Chi-square test or Student's *T*-test, *P* > 0.05, Table 1). Univariate Cox regression analysis was used to evaluate the association between survival and expression level of each TF and lncRNA. The risk score for each patient was calculated according to the linear combination of expression values weighted by the coefficient from the univariate Cox regression analysis:

(3) $$\begin{array}{r} RiskScore=\overset{n}{\underset{i=1}{\sum }}r_{i}Exp\left(i\right) \end{array}$$

Where *r*_*i*_ is the Cox regression coefficient of a TF or lncRNA node in the training set, n is the number of nodes in the clique. *Exp*(*i*) is the expression value of node i. The median risk score was used as the cut-off to divide patients into high- and low-risk groups. The patients in the high-risk group were expected to have poor survival outcomes. Conversely, the patients in the low-risk group were expected to have good survival outcomes.

### Functional Analysis

Functional annotation for the TFs and lncRNAs was performed using the Enrich web based tool (http://amp.pharm.mssm.edu/Enrichr/) (Kuleshov et al., 2016), which performs a comprehensive gene set enrichment analysis based on 180,184 annotated gene sets from 102 gene set libraries. The fuzzy *P*-value enrichment score can be calculated by decomposing the null distribution.

### Statistical Analysis

Statistical analyses were performed based on R 3.1.0 framework. Kaplan-Meier survival curves were plotted for different groups of patients, and statistical significance was assessed using the log-rank test (*P* < 0.05). In two groups of OC patients, significant differences in several OC clinicopathologic factors such as stage, age, histological grade type, and survival status were considered and assessed using the Chi-square test or Student's *t*-test (*P* < 0.05). The K-means clustering method was used to classify the TF-lncRNA relationships into different groups based on the regulatory activity across different OC stages. The Jaccard coefficient was used to evaluate the similarity between two TF-lncRNA cliques. It is a statistical method for comparing similarity and diversity of two datasets. For two datasets X and Y, the Jaccard coefficient is defined as the size of the intersection divided by the size of the union of the sample sets:

(4) $$\begin{array}{r} J\left(X,Y\right)=\frac{\left|X\cap Y\right|}{\left|X\cup Y\right|} \end{array}$$

## Results and Discussion

### Construction of Stage-Specific TF-lncRNA Regulatory Networks in OC Development

**Figure 1:**
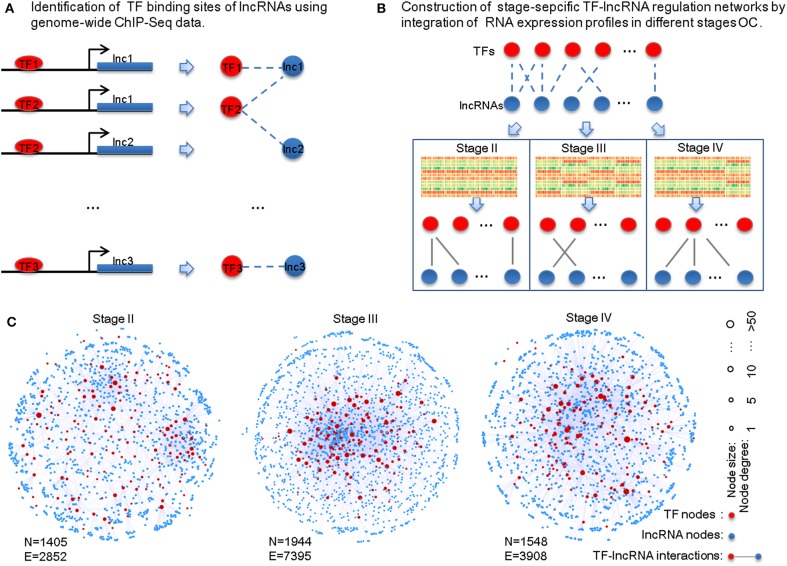
Construction of the stage-specific TF-lncRNA regulatory networks in OC. **(A)** Identification of conserved transcription factor binding sites on lncRNAs from ChIP-Seq datasets. **(B)** Identification of stage-specific TF-lncRNA relationships in different OC stages based on co-expression analysis. **(C)** Network illustration of the three stage-specific TF-lncRNA regulatory networks. N, nodes; E, edges.

To identify potential TF-lncRNA regulations, we used an integrated pipeline, which was developed and applied in our previous studies (Ning et al., 2014, 2016). This pipeline integrates conserved transcription factor binding sites based on multiz46way alignments of UCSC (Karolchik et al., 2003) and ChIP-Seq datasets in different cell lines and tissues from the ENCODE project (Wellcome Trust Case Control Consortium, 2007). Consequently, we obtained 58,119 potential TF-lncRNA interactions among 151 TFs and 3,981 lncRNAs (Figure 1A). Considering that predictive binding sites of TFs do not directly imply their actual regulation of lncRNAs in certain conditions, exploring TF regulation of lncRNAs through co-expression analysis can offer useful information to identify active TF-lncRNA relationships in different OC stages. Pearson correlation coefficients were calculated for each potential TF-lncRNA pair based on their expression values at different stages (Figure 1B). We used Pearson coefficient >0 and FDR < 0.05 as the thresholds to identify links between TFs and lncRNAs in the regulatory networks. Finally, stage-specific TF-lncRNA regulatory networks were constructed (Figure 1C). Specifically, 2,852 edges between 144 TFs and 1,261 lncRNAs, 7,395 edges between 147 TFs and 1,797 lncRNAs and 3,908 edges between 145 TFs and 1,403 lncRNAs were constructed for stages II, III, and IV OC patients, respectively. Although 58,119 potential TF-lncRNA relationships were identified based on initial sequence binding analysis, only a fraction (4.91–6.72%) of these regulating edges were actively constructed in each stage. The different sizes of these stage-specific networks indicate the heterogeneity of TF-lncRNA interactions in the development of OC.

### Common and Specific Features of Stage-Specific TF-lncRNA Regulatory Networks

**Figure 2:**
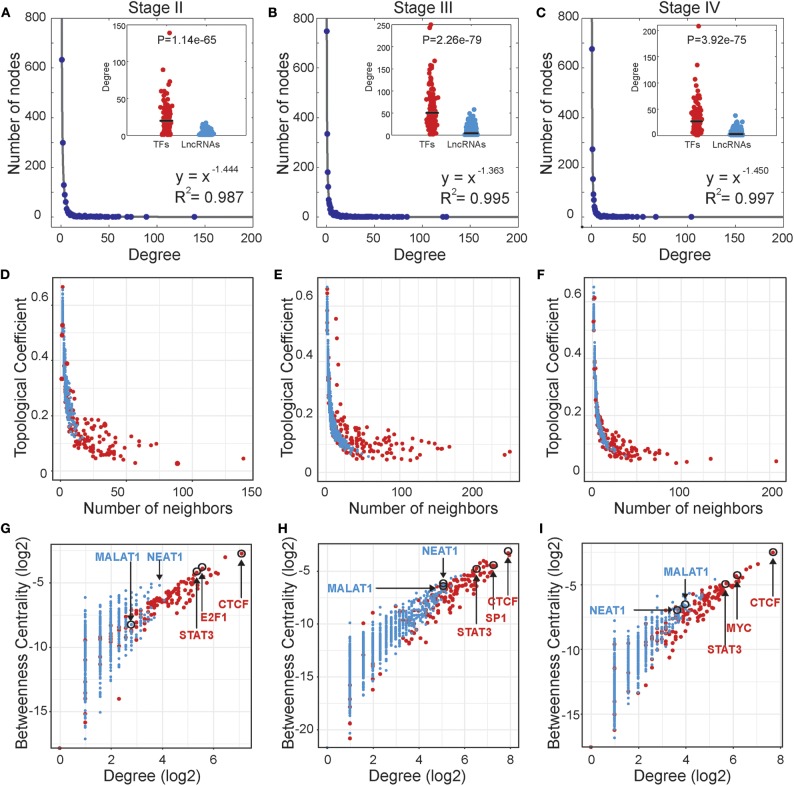
Topological properties of stage-specific TF-lncRNA regulatory networks. **(A–C)** The degree distributions of nodes in the three networks. The TF nodes have significantly higher degree values than lncRNA nodes in each stage. **(D–F)** The topological coefficients decreased with the increase in number of neighbors. **(G–I)** Some well-known OC risk TFs and lncRNAs can be found in the TF-lncRNA networks and exhibit higher values of betweenness centrality and degree.

Based on the stage-specific TF-lncRNA regulatory networks, we performed an analysis of the network structures and topological properties. We found that more than 95% of TFs regulating at least two lncRNAs and more than 50% of the lncRNAs were co-regulated by two or more TFs. Most TFs and lncRNAs were connected and participated in the major component (the largest connected subnetwork) of the regulatory networks. We examined the degree distributions of both TF and lncRNA nodes and observed power-law distributions in all stage-specific networks (Figures 2A–C). These results reveal that the three TF-lncRNA regulatory networks are similar to classic biological networks and well-characterized by a core set of regulating principles in structure (Nacher and Akutsu, 2007). TF nodes had significantly higher degree values than lncRNA nodes in each stage, indicating the complicated combinations in terms of TF co-regulation and lncRNA multiplicity (Figures 2A–C). The negative association between topological coefficients and number of neighbors for TF and lncRNA nodes explains that hubs are rather exclusive with rare common neighbors compared to individual nodes with fewer links (Figures 2D–F). This observation indicates that TF-lncRNA regulatory networks have hierarchical modularity and subnetworks (Ning et al., 2016).

In addition, some well-known TFs and lncRNAs were found in the TF-lncRNA networks and exhibited higher values of betweenness centrality and degree (Figures 2G–I). The higher betweenness centrality indicates that the node is a bottleneck site and acts as a bridge connecting different parts of the network (Wang et al., 2015). The higher degree indicates that the node is a hub site that participates in additional regulating interactions. For example, STAT3 is a well-identified oncogene that promotes invasion and metastasis of OC (Pradeep et al., 2015). CTCF expression is closely associated with DNA hypomethylation and confers poor prognosis on OC patients (Woloszynska-Read et al., 2011). E2F1, the most classic member of the E2F family, has been determined to play dual functions and serve as a useful prognostic indicator in OC (Zhan et al., 2016). The lncRNA MALAT1 has been found to promote proliferation and metastasis in epithelial ovarian cancer via the PI3K-AKT pathway (Li et al., 2016). Overexpression of NEAT1 is associated with poor OC prognosis and promotes tumor growth and progression (Chai et al., 2016). Furthermore, we found that some TFs tend to act as temporal-hub and bottleneck nodes in certain stages of OC progression, such as SP1 in stage III and MYC in stage IV.

### The Dynamic Activity Profile of TF-lncRNA Regulations

**Figure 3:**
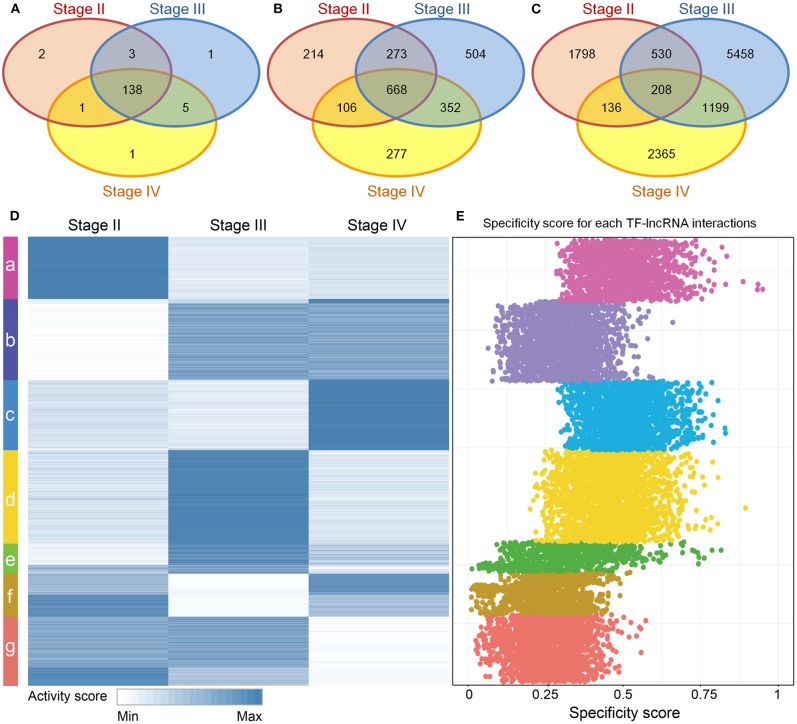
Dynamic TF-lncRNA interactions across the progression of OC. **(A–C)** Venn diagrams showing the overlaps of TFs, lncRNAs and their regulation in different stages. TFs in different stages of OC regulate different target lncRNAs. **(D)** The activity profile for TF-lncRNA relationships across different stages of OC. The co-expression coefficients for TF-lncRNA pairs in each stage of OC were used as activity scores. Clustering analysis revealed that different groups of TF-lncRNA relationships were apparently activated in one or more stages of OC. **(E)** The corresponding specificity scores were plotted. The groups a, c, d, and e have higher specificity scores than other groups, indicating these groups are stage specific.

Although stage-specific networks share common topological properties, the TF-lncRNA regulatory interactions may change in different OC stages. To evaluate the proportion of common and specific TF-lncRNA relationships during OC progression, we explored the overlaps of TFs, lncRNAs and their regulatory relationships among three stage-specific networks (Figure S2). More than 90% TFs were common to all three stages of OC (Figure 3A) while only about 28% lncRNAs were common to all three stages, indicating that lncRNAs were more temporally-specific than TFs (Figure 3B). Only 1.8% of TF-lncRNA relationships were retained in all three stages. Most of them were involved in only one stage (Figure 3C). These results indicate that TFs in different OC stages tend to selectively regulate different targets, similar to a previous conclusion on glioma (Li et al., 2015), which may help us interpret the pathological divergence of OC progression.

To provide an overview of all possible TF-lncRNA relationships and their dynamic regulatory status, we built an activity profile for TF-lncRNA relationships across different OC stages (Figure 3D). The co-expression coefficients for a TF-lncRNA pair in all stages were normalized and used as the activity scores (Materials and Methods). This strategy has been applied in our previous work to evaluate lncRNA-miRNA-mRNA competing activity across 12 types of cancers (Wang et al., 2015). Based on activity scores, these TF-lncRNA interactions were grouped by the K-means clustering method. Different groups of TF-lncRNA interactions were apparently activated at one or more stages. To evaluate the specificity of each group, we calculated the specificity score for every TF-lncRNA relationship (Materials and Methods). The corresponding specificity scores were plotted in Figure 3E. The groups a, c, d, and e have higher specificity scores than other groups, indicating these groups were more stage-specific. Other groups such as b, f and g were less specific and were involved in two or three stages. For example, lncRNA NEAT1 was associated with poor prognosis and was highly upregulated in late OC stages (III and IV) (Chai et al., 2016). We found that NEAT1 was involved in group b, which was specific to stages III and IV, and regulated by NR3C1, FOSL1, and CEBPD. These genes have been found to be associated with OC progression (Huang et al., 2007; Simpkins et al., 2012; Lau et al., 2014). The mean specificity score of all TF-lncRNA regulations was 0.38, and more than 94% of these interactions had specificity scores >0.15, which is a threshold indicating a housekeeping gene (Yanai et al., 2005) (Figure S3). These results reveal a high degree of specificity for TF-lncRNA interactions in different stages of OC.

### Functional Characterization of Stage-Specific TF-lncRNA Relationships

**Figure 4:**
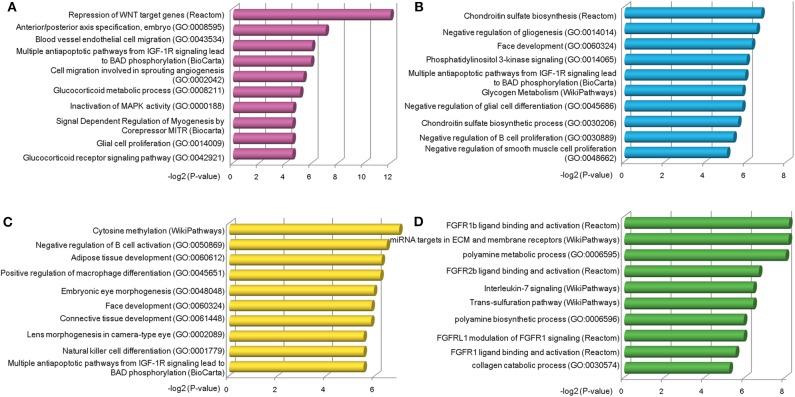
Functional analysis of stage-specific TF-lncRNA regulatory groups. **(A)** Group a was found to be associated with Repression of WNT target genes and other OC-related pathways. **(B)** Group c was found to be associated with chondroitin sulfate biosynthesis pathway and process (GO:0030206). **(C)** A series of immune cell activation and differentiation processes were found in group d. **(D)** Group e was associated with FGFR ligand binding and activation pathways.

Next, we explored the functions of lncRNAs by using Enrichr web based tool (Chen et al., 2013; Kuleshov et al., 2016), which performs a comprehensive gene set enrichment analysis based on different functional contexts. For lncRNAs in each group, we found a series of common and specific functions across different OC stages (Table S7). For example, group a was specifically activated in stage II OC patients. This group was found to be associated with Repression of WNT target genes, multiple antiapoptotic pathways from IGF-1R signaling which lead to BAD phosphorylation and some other OC-related pathways (Figure 4A). The WNT pathway has been found to be involved in OC progression (Yoshioka et al., 2012) and be associated with clinical outcomes (Reinartz et al., 2016). Via modulation of BAD phosphorylation, the BAD apoptosis pathway influences chemosensitivity and overall survival of OC (Marchion et al., 2011). This pathway was enriched in groups c and d. LncRNAs in group a were found to be involved in tumor amplification-related biological processes such as cell migration and proliferation. We also found that lncRNAs in group a were associated with inactivation of MAPK activity (GO:0000188), which is a critical pathway for human cancer cell survival, dissemination, and drug therapy (Zhang et al., 2015). These functional annotation results reveal that lncRNAs in group a are involved in uncontrolled tumor cell growth and processes of development at an early OC stage. For group c, which was specific to stage IV patients, the chondroitin sulfate biosynthesis pathway and process (GO:0030206) were found in the enriched functional list (Figure 4B). Chondroitin sulfate is a natural product of tumor fibrosis and is highly expressed in fibrotic OC tissue (Nash et al., 2002). A previous study revealed that the stroma of late OC stage showed significantly increased expression of 4,6-disulfated chondroitin sulfate, which is an independent predictor for progression-free survival in OC (Vallen et al., 2012). For group d, a series of immune cell activation and differentiation processes, including regulation of B-cell activation and macrophage differentiation, were enriched (Figure 4C). It has been well-documented that the immune cell system is a natural defense against tumor cells (Nogrady, 2014) and associated with OC patients' clinical outcomes (Curiel et al., 2004). Group e was found to be related with FGFR ligand binding and activation pathways (Figure 4D), which are associated with cisplatin sensitivity in OC clinical treatment (Cole et al., 2010).

### Identification of Dynamic TF-lncRNA Regulatory Cliques Associating With Cancer Hallmarks

**Figure 5:**
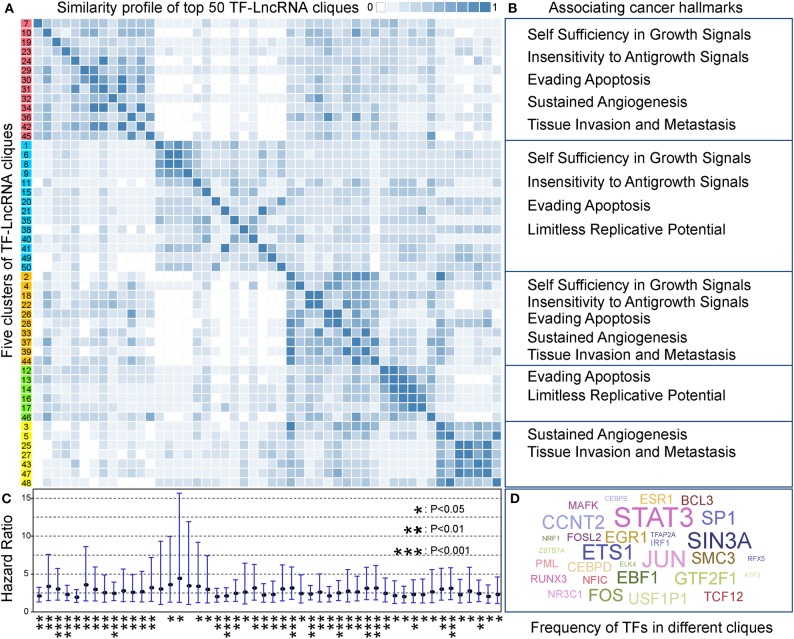
TF-lncRNA regulatory cliques are associated with cancer hallmarks. **(A)** Clustering profile of the top 50 cliques based on their similarity. **(B)** Different clusters of cliques were associated with a broad range of cancer hallmarks. **(C)** The hazard ratio and 95% confidence interval for each of the 50 TF-lncRNA cliques based on Cox regression analysis. Most of the cliques (48 of 50) were significantly associated with patient prognosis (*P* < 0.05). *P < 0.05, **P < 0.01, and ***P < 0.001. **(D)** A world cloud map indicating TF frequency in the 50 cliques. Some well-known cancer prognostic genes such as STAT3, ETS1, and FOS were frequently involved in different cliques.

In TF-lncRNA regulatory networks, one TF can regulate different lncRNAs, while one lncRNA can be regulated by different TFs during tumor progression. Thus, partial network cliques can provide more detailed information of cross-talk between different TFs and lncRNAs. Topological analysis revealed that the TF-lncRNA regulatory networks have hierarchical modularity (Figures 2D–F). We then identified TF-lncRNA regulating cliques using the biclique method, which is an R package for enumerating maximal complete bipartite graphs (Zhang et al., 2014). In total, 4,886 TF-lncRNA cliques were identified, in which the number of nodes ranges from 5 to 220 with an average of 11.74 nodes per clique. Considering that 4,886 cliques is a large number for further analysis and that some cliques may have overlaps with each other, we ranked these cliques in descending order based on the mean specificity scores (Materials and Methods). The top 50 (~1% of 4,886) cliques, which were highly specific across different OC stages, were retained for further analysis. A clustering profile of these top 50 cliques was built based on the similarity between every two clique pairs (Figure 5A). Jaccard coefficient was used to evaluate the similarity between two cliques. We found that these cliques were generally grouped into five clusters. In each cluster, cliques were similar to each other for sharing common TFs and lncRNAs. Further, we found that different clusters of cliques were associating with a broad range of cancer hallmarks (Figure 5B), which have been determined to promote tumor growth and metastasis (Hanahan and Weinberg, 2011). For example, the hallmarks including “Self Sufficiency in Growth Signals,” “Tissue Invasion and Metastasis,” and “Evading Apoptosis” were the most highly enriched processes, indicating the rapid growth and high metastasis rates of OC (Nieman et al., 2011).

We built a risk model to evaluate the prognostic efficiency of these 50 highly specific TF-lncRNA cliques (Materials and Methods). The hazard ratio and corresponding confidence interval for each clique were shown in Figure 5C. We found most cliques (48 of 50) were significantly associating with patient prognosis (*P* < 0.05). Among them, 10 cliques were highly significant with *P* < 0.001 (Table S8). To illustrate which TF were involved in these cliques, a world cloud map indicating TF frequency was constructed (Figure 5D). We found that some well-known cancer prognostic genes, such as STAT3, ETS1, and FOS, were frequently involved in different cliques. These findings reveal that highly specific TF-lncRNA cliques play critical roles in OC tumorigenesis and may be potential prognostic markers.

### Survival Analysis of a STAT3-FOS-Regulating TF-lncRNA Clique

**Figure 6:**
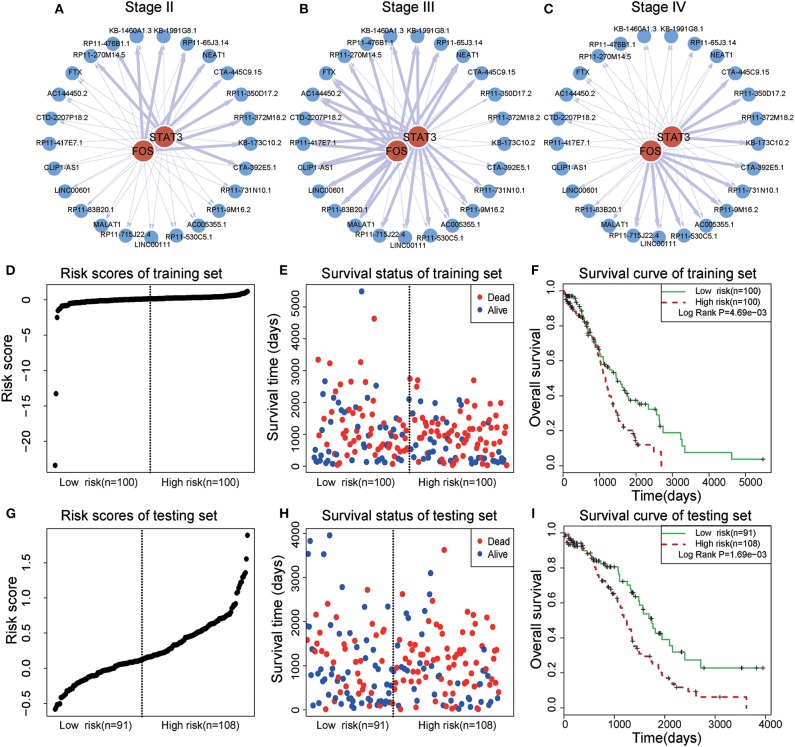
Survival analysis of a STAT3-FOS-regulating TF-lncRNA clique. **(A–C)** Illustration of TF-lncRNA regulatory relationships in different OC stages. The TFs STAT3 and FOS selectively regulate different target lncRNAs. **(D,E)** The risk scores and survival status of this clique in the training dataset. **(F)** Kaplan-Meier survival curves revealed significant difference in survival between the two groups of patients in the training dataset. The high-risk group consisted of patients with higher risk scores and shorter survival time. **(G,H)** Risk scores and survival status of this clique in the testing dataset. **(I)** Kaplan-Meier survival curves for two groups in the testing dataset using the same risk score threshold as the training set. There was significant difference between high- and low-risk groups.

In a TF-lncRNA clique (clique 32 in Figure 5A), TFs STAT3 and FOS were found to selectively regulate different target lncRNAs, including two known OC-risk lncRNAs, MALAT1, and NEAT1 (Figures 6A–C). Both of MALAT1 and NEAT1 regulate cell proliferation and apoptosis of OC (Yong et al., 2018; Sun et al., 2019). In the above Cox regression analysis, this clique was found to be significantly associated with prognosis (*P* = 2.78e-4). To further evaluate the prognostic efficiency, the 399 OC patients were randomly divided into two groups used as training (*n* = 200) and testing (*n* = 199) datasets (Table 1). There were no significant differences in the clinical characteristics between the two groups (*P* > 0.05). In the training dataset, the median risk score was used as a threshold to divide patients into two sub-groups (Figures 6D–E). Kaplan-Meier survival analysis revealed significant difference in survival between the two sub-groups of patients in the training dataset (Figure 6F, *P* = 4.69e-3). The high-risk group consisted of patients with higher risk scores and lower survival time. Furthermore, we investigated the prognostic efficiency of this STAT3-FOS clique in the testing dataset using the same risk score threshold from training set. Using this strategy, the testing patients could also be significantly divided into high- and low-risk sub-groups (Figures 6G–I, *P* = 1.69e-3).

To further evaluate the prognostic independence of STAT3-FOS clique, the prognostic association with other known clinical and pathological risk factors was estimated by univariate and multivariate analysis. Several OC clinicopathologic factors, such as stage, age, and histological grade type were taken into consideration. As expected, in addition to patient age (Table 2, *P* = 0.01), which is already a known OC risk factor (Ferrero et al., 2017), the STAT3-FOS clique was significantly associated with survival in the univariate (Table 2, *P* = 6.22e-4) and multivariate analysis (Table 2, *P* = 1.05e-3), indicating the potential independent prognostic ability of this clique.

**Table 2:** Univariate and multivariate analysis of clinicopathological factors and the STAT3-FOS clique in 399 OC patients.

**Variables**	**Univariate analysis**			**Multivariate analysis**		
	**HR (95% CI)**	**Coefficient**	***P***	**HR (95% CI)**	**Coefficient**	***P***
Stage	1.31 (0.98–1.75)	0.27	0.07	1.26 (0.93–1.70)	0.23	0.13
Age	1.02 (1.00–1.03)	0.02	**0.01**	1.02 (1.00–1.03)	0.02	**0.02**
Histological grade	1.06 (0.81–1.39)	0.06	0.69	1.04 (0.80–1.36)	0.04	0.76
STAT3-FOS clique	2.58 (1.50–4.43)	0.95	**6.22e-4**	2.50 (1.45–4.32)	0.92	**1.00e-3**

*The bold values are representing significant factors (P-values < 0.05)*.

### Further Validation of Prognosis in Another Two Independent Datasets

**Figure 7:**
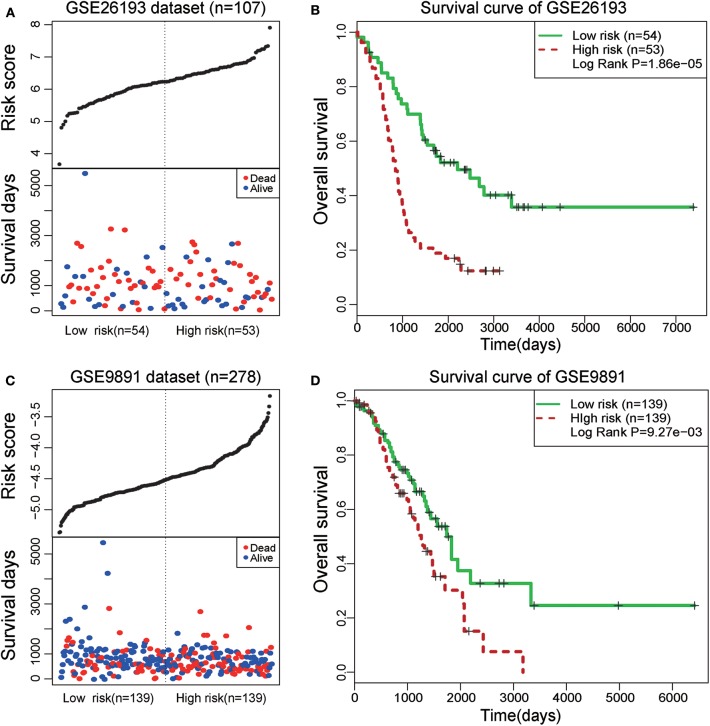
Survival analysis of STAT3-FOS-regulating TF-lncRNA clique in another two-independent dataset. **(A)** The risk scores and survival status of this clique in the GSE26193 dataset. **(B)** Kaplan-Meier survival curves revealed significant difference in survival between the two groups of patients in the GSE26193 dataset. **(C)** The risk scores and survival status of this clique in the GSE9891 dataset. **(D)** Kaplan-Meier survival curves revealed significant difference in survival between the two groups of patients in the GSE9891 dataset.

To further test the prognostic efficiency and application scope of the STAT3-FOS clique, we collected another two independent OC datasets (GSE26193 and GSE9891) and applied the same survival risk model to them. Univariate Cox regression analysis indicated it was significantly associated with patients' survival in the dataset of GSE26193 (HR = 2.51, 95% CI = 1.65–3.84, *P* = 1.97e-5) and GSE9891 (HR = 2.04, 95% CI = 1.310–3.18, *P* = 1.65e-3). Kaplan-Meier survival analysis indicated it could significantly divide the 107 patients of GSE26193 dataset into high- and low-risk sub-groups (Figures 7A,B, *P* = 1.86e-5). Further, this TF-lncRNA clique could also significantly divide the 278 patients of GSE9891 into two sub-groups with different survivals (Figures 7C,D, *P* = 9.27e-3). These results are consistent with the observation in the TCGA dataset. Although some lncRNAs in this clique were not covered by these microarray datasets, the risk model could also predict high- and low-risk groups of patients only based on partial TF and lncRNA members, indicating the robustness of prognostic efficiency for this clique. These results indicate that the STAT3-FOS clique could be used as a potential prognostic factor for OC.

## Conclusions

In recent years, many studies have been carried out to characterize lncRNAs functions based on the general assumption that lncRNAs are key regulators of biological processes. Although these studies have successfully characterized the roles of lncRNAs, little is known about the upstream regulators of lncRNAs. In biological processes and signaling pathways, lncRNAs may be regulated by other regulators such as TFs. In production processes of lncRNAs, TFs play key roles in controlling lncRNA expression through sequence-specific binding sites on lncRNA transcripts. The differential expression of TFs and their downstream lncRNA targets have demonstrated association with progression of multiple types of diseases (Dong et al., 2015).

The pathological development of OC is a complex progression that depends on multiple alterations of coding and non-coding genes. Although our understanding of OC has increased, the precise regulatory mechanisms underlying this complex disease are still not fully known. Therefore, it is important to uncover the regulating events during OC progression and identify reliable markers for predicting clinical outcomes. To address this issue, we constructed stage-specific TF-lncRNA regulatory networks based on 399 ovarian serous cystadenocarcinoma patients with different stages from TCGA. Our study indicated that all three stage-specific TF-lncRNA regulatory networks exhibited common network structures and specific topologies of risk TFs and lncRNAs. A TF-lncRNA activity profile revealed that TFs were highly stage-selective in regulating lncRNAs. Functional analysis indicated that groups of TF-lncRNA interactions tend to be involved in specific pathological processes in OC development. Based on network clique analysis, we found that TF-lncRNA bicliques were associating with several cancer hallmarks. These hallmarks provide a framework for understanding the remarkable diversity of cancers. For example, the hallmarks including “Self Sufficiency in Growth Signals,” “Tissue Invasion and Metastasis,” and “Evading Apoptosis” were the most highly enriched processes. Survival analysis of TF-lncRNA bicliques indicated the potential role for predicting OC prognosis. Although these results are interesting and meaningful, we still lack the biological experiments required for further validation. We will continue to investigate the biological mechanisms governing how lncRNAs regulate cell cycle genes during carcinogenesis in our future work.

## Data Availability Statement

All data included in this study are available upon request by contact with the corresponding author.

## Author Contributions

SN and PW conceived and designed the experiments. QG, JW, and YG analyzed data. XL and YH collected the data. JW and YG validated the method and data. QG wrote this manuscript. All authors read and approved the final manuscript.

### Conflict of Interest

The authors declare that the research was conducted in the absence of any commercial or financial relationships that could be construed as a potential conflict of interest.
