# Supplementary material for: Dynamic TF-lncRNA Regulatory Networks Revealed Prognostic Signatures in the Development of Ovarian Cancer
Source: Front Bioeng Biotechnol. 2020 May 13;8:460. doi: 10.3389/fbioe.2020.00460 (PMC7237576; doi:10.3389/fbioe.2020.00460)
Supplement: Supplementary file 7 [file Table_7.DOCX]

**Table S7.** Top 10 siginficantly enriched biological processes and pathways for different groups of TF-lncRNA relationships.

| **Groups** | **Enriched biological terms and pathways** | **P-values** |
| --- | --- | --- |
|  | Repression of WNT target genes Homo sapiens R-HSA-4641265 | 2.07E-04 |
|  | anterior/posterior axis specification, embryo (GO:0008595) | 7.50E-03 |
|  | blood vessel endothelial cell migration (GO:0043534) | 1.55E-02 |
|  | Multiple antiapoptotic pathways from IGF-1R signaling lead to BAD phosphorylation Homo sapiens h igf1rPathway | 1.63E-02 |
| group a | cell migration involved in sprouting angiogenesis (GO:0002042) | 2.43E-02 |
|  | glucocorticoid metabolic process (GO:0008211) | 2.89E-02 |
|  | inactivation of MAPK activity (GO:0000188) | 4.21E-02 |
|  | glucocorticoid receptor signaling pathway (GO:0042921) | 4.32E-02 |
|  | glial cell proliferation (GO:0014009) | 4.32E-02 |
|  | Signal Dependent Regulation of Myogenesis by Corepressor MITR Homo sapiens h MITRPathway | 4.32E-02 |
|  | Tyrosine metabolism_Homo sapiens_hsa00350 | 7.25E-03 |
|  | RAF-independent MAPK1/3 activation_Homo sapiens_R-HSA-112409 | 7.95E-03 |
|  | FGFRL1 modulation of FGFR1 signaling_Homo sapiens_R-HSA-5658623 | 9.58E-03 |
|  | Phenylalanine, tyrosine and tryptophan biosynthesis_Homo sapiens_hsa00400 | 1.16E-02 |
| group b | miRNA targets in ECM and membrane receptors_Homo sapiens_WP2911 | 1.41E-02 |
|  | positive regulation of cell migration involved in sprouting angiogenesis | 1.60E-02 |
|  | FGFR1b ligand binding and activation_Homo sapiens_R-HSA-190370 | 1.69E-02 |
|  | Phosphorylation of Emi1_Homo sapiens_R-HSA-176417 | 1.69E-02 |
|  | glomerular visceral epithelial cell fate commitment | 1.69E-02 |
|  | mesonephric glomerular visceral epithelial cell differentiation | 1.69E-02 |
|  | Chondroitin sulfate biosynthesis Homo sapiens R-HSA-2022870 | 9.33E-03 |
|  | negative regulation of gliogenesis (GO:0014014) | 1.09E-02 |
|  | face development (GO:0060324) | 1.29E-02 |
|  | phosphatidylinositol 3-kinase signaling (GO:0014065) | 1.54E-02 |
| group c | Multiple antiapoptotic pathways from IGF-1R signaling lead to BAD phosphorylation Homo sapiens h igf1rPathway | 1.62E-02 |
|  | Glycogen Metabolism Homo sapiens WP500 | 1.78E-02 |
|  | negative regulation of glial cell differentiation (GO:0045686) | 1.79E-02 |
|  | chondroitin sulfate biosynthetic process (GO:0030206) | 2.06E-02 |
|  | negative regulation of B cell proliferation (GO:0030889) | 2.42E-02 |
|  | negative regulation of smooth muscle cell proliferation (GO:0048662) | 3.01E-02 |
|  | Cytosine methylation Homo sapiens WP3585 | 7.32E-03 |
|  | negative regulation of B cell activation (GO:0050869) | 1.12E-02 |
|  | adipose tissue development (GO:0060612) | 1.29E-02 |
|  | positive regulation of macrophage differentiation (GO:0045651) | 1.34E-02 |
| group d | embryonic eye morphogenesis (GO:0048048) | 1.61E-02 |
|  | face development (GO:0060324) | 1.72E-02 |
|  | connective tissue development (GO:0061448) | 1.74E-02 |
|  | lens morphogenesis in camera-type eye (GO:0002089) | 2.16E-02 |
|  | natural killer cell differentiation (GO:0001779) | 2.16E-02 |
|  | Multiple antiapoptotic pathways from IGF-1R signaling lead to BAD phosphorylation Homo sapiens h igf1rPathway | 2.16E-02 |
|  | FGFR1b ligand binding and activation Homo sapiens R-HSA-190370 | 3.34E-03 |
|  | miRNA targets in ECM and membrane receptors Homo sapiens WP2911 | 3.45E-03 |
|  | polyamine metabolic process (GO:0006595) | 3.81E-03 |
|  | FGFR2b ligand binding and activation Homo sapiens R-HSA-190377 | 9.62E-03 |
| group e | Trans-sulfuration pathway Homo sapiens WP2333 | 1.16E-02 |
|  | Interleukin-7 signaling Homo sapiens R-HSA-1266695 | 1.16E-02 |
|  | FGFRL1 modulation of FGFR1 signaling Homo sapiens R-HSA-5658623 | 1.62E-02 |
|  | polyamine biosynthetic process (GO:0006596) | 1.62E-02 |
|  | FGFR1 ligand binding and activation Homo sapiens R-HSA-190242 | 2.14E-02 |
|  | collagen catabolic process (GO:0030574) | 2.65E-02 |
|  | mammary gland development (GO:0030879) | 3.20E-03 |
|  | glutamine family amino acid catabolic process (GO:0009065) | 5.29E-03 |
|  | Phenylalanine, tyrosine and tryptophan biosynthesis Homo sapiens hsa00400 | 8.46E-03 |
|  | Glycosphingolipid biosynthesis - ganglio series Homo sapiens hsa00604 | 9.34E-03 |
| group f | renal system process involved in regulation of blood volume (GO:0001977) | 9.34E-03 |
|  | 2-oxoglutarate metabolic process (GO:0006103) | 1.12E-02 |
|  | Phenylalanine metabolism Homo sapiens hsa00360 | 1.34E-02 |
|  | 2-Oxocarboxylic acid metabolism Homo sapiens hsa01210 | 1.34E-02 |
|  | apoptotic process involved in morphogenesis (GO:0060561) | 1.34E-02 |
|  | Tyrosine metabolism Homo sapiens hsa00350 | 2.01E-02 |
|  | embryonic camera-type eye morphogenesis | 2.33E-04 |
|  | embryonic camera-type eye development | 6.82E-04 |
|  | Cytosine methylation_Homo sapiens_WP3585 | 3.12E-03 |
|  | protein O-linked glycosylation via serine | 6.40E-03 |
| group g | protein O-linked glycosylation via threonine | 7.25E-03 |
|  | FGFRL1 modulation of FGFR1 signaling_Homo sapiens_R-HSA-5658623 | 9.58E-03 |
|  | D-glutamate metabolic process | 9.58E-03 |
|  | tetrapyrrole biosynthetic process from glutamate | 9.58E-03 |
|  | Phenylalanine, tyrosine and tryptophan biosynthesis_Homo sapiens_hsa00400 | 1.16E-02 |
|  | L-arabinose catabolic process to 2-oxoglutarate | 1.19E-02 |
