# Supplementary material for: Dynamic TF-lncRNA Regulatory Networks Revealed Prognostic Signatures in the Development of Ovarian Cancer
Source: Front Bioeng Biotechnol. 2020 May 13;8:460. doi: 10.3389/fbioe.2020.00460 (PMC7237576; doi:10.3389/fbioe.2020.00460)
Supplement: Supplementary file 8 [file Table_8.DOCX]

**Table S8.** Univariate analysis Cox regression analysis of the top 50 specific TF-lncRNA cliques.

| **Rank** | **HR (95%CI)** | **Coefficient** | **P** |
| --- | --- | --- | --- |
| 1 | 2.25(0.84-6.08) | 0.81 | 1.08E-01 |
| 2 | 3.85(1.58-9.38) | 1.35 | 2.99E-03 |
| 3 | 2.55(1.49-4.36) | 0.94 | 6.61E-04 |
| 4 | 2.97(1.40-6.27) | 1.09 | 4.46E-03 |
| 5 | 2.13(1.38-3.29) | 0.76 | 6.40E-04 |
| 6 | 3.33(0.60-18.38) | 1.20 | 1.68E-01 |
| 7 | 2.18(0.80-5.95) | 0.78 | 1.26E-01 |
| 8 | 3.97(0.47-33.30) | 1.38 | 2.03E-01 |
| 9 | 3.45(0.73-16.28) | 1.24 | 1.17E-01 |
| 10 | 1.82(0.94-3.52) | 0.60 | 7.65E-02 |
| 11 | 2.50(1.03-6.06) | 0.92 | 4.27E-02 |
| 12 | 2.56(1.21-5.41) | 0.94 | 1.38E-02 |
| 13 | 2.00(1.13-3.55) | 0.69 | 1.79E-02 |
| 14 | 2.11(1.16-3.83) | 0.75 | 1.47E-02 |
| 15 | 3.25(0.36-29.44) | 1.18 | 2.95E-01 |
| 16 | 2.72(1.05-7.01) | 1.00 | 3.89E-02 |
| 17 | 3.19(0.71-14.26) | 1.16 | 1.29E-01 |
| 18 | 2.52(1.49-4.26) | 0.93 | 5.31E-04 |
| 19 | 2.38(1.37-4.14) | 0.87 | 2.07E-03 |
| 20 | 2.59(1.62-4.14) | 0.95 | 7.33E-05 |
| 21 | 2.60(1.32-5.12) | 0.96 | 5.72E-03 |
| 22 | 2.06(1.16-3.66) | 0.72 | 1.43E-02 |
| 23 | 2.44(1.09-5.46) | 0.89 | 2.99E-02 |
| 24 | 2.66(1.20-5.92) | 0.98 | 1.64E-02 |
| 25 | 2.66(1.50-4.71) | 0.98 | 8.08E-04 |
| 26 | 3.00(1.27-7.08) | 1.10 | 1.23E-02 |
| 27 | 2.82(1.45-5.51) | 1.04 | 2.36E-03 |
| 28 | 2.41(1.03-5.62) | 0.88 | 4.25E-02 |
| 29 | 2.38(0.99-5.71) | 0.87 | 5.28E-02 |
| 30 | 1.81(0.96-3.39) | 0.59 | 6.49E-02 |
| 31 | 2.77(1.38-5.54) | 1.02 | 4.03E-03 |
| 32 | 2.58(1.50-4.43) | 0.95 | 6.22E-04 |
| 33 | 2.17(1.12-4.21) | 0.78 | 2.15E-02 |
| 34 | 2.27(1.25-4.13) | 0.82 | 7.44E-03 |
| 35 | 2.53(1.12-5.72) | 0.93 | 2.59E-02 |
| 36 | 3.27(1.23-8.70) | 1.18 | 1.78E-02 |
| 37 | 3.32(1.46-7.59) | 1.20 | 4.37E-03 |
| 38 | 2.01(1.08-3.75) | 0.70 | 2.79E-02 |
| 39 | 3.90(1.45-10.51) | 1.36 | 7.08E-03 |
| 40 | 2.92(1.51-5.65) | 1.07 | 1.44E-03 |
| 41 | 2.73(1.05-7.10) | 1.01 | 3.90E-02 |
| 42 | 2.59(1.33-5.04) | 0.95 | 4.96E-03 |
| 43 | 3.22(1.73-5.99) | 1.17 | 2.23E-04 |
| 44 | 2.58(1.27-5.23) | 0.95 | 8.70E-03 |
| 45 | 2.65(1.08-6.55) | 0.98 | 3.41E-02 |
| 46 | 2.17(1.11-4.22) | 0.77 | 2.30E-02 |
| 47 | 2.74(1.63-4.60) | 1.01 | 1.33E-04 |
| 48 | 1.85(1.23-2.80) | 0.62 | 3.33E-03 |
| 49 | 2.64(0.88-7.92) | 0.97 | 8.26E-02 |
| 50 | 3.20(1.54-6.65) | 1.16 | 1.89E-03 |
