# Supplementary material for: Dynamic TF-lncRNA Regulatory Networks Revealed Prognostic Signatures in the Development of Ovarian Cancer
Source: Front Bioeng Biotechnol. 2020 May 13;8:460. doi: 10.3389/fbioe.2020.00460 (PMC7237576; doi:10.3389/fbioe.2020.00460)
Supplement: Supplementary file 9 [file Data_Sheet_1.PDF]

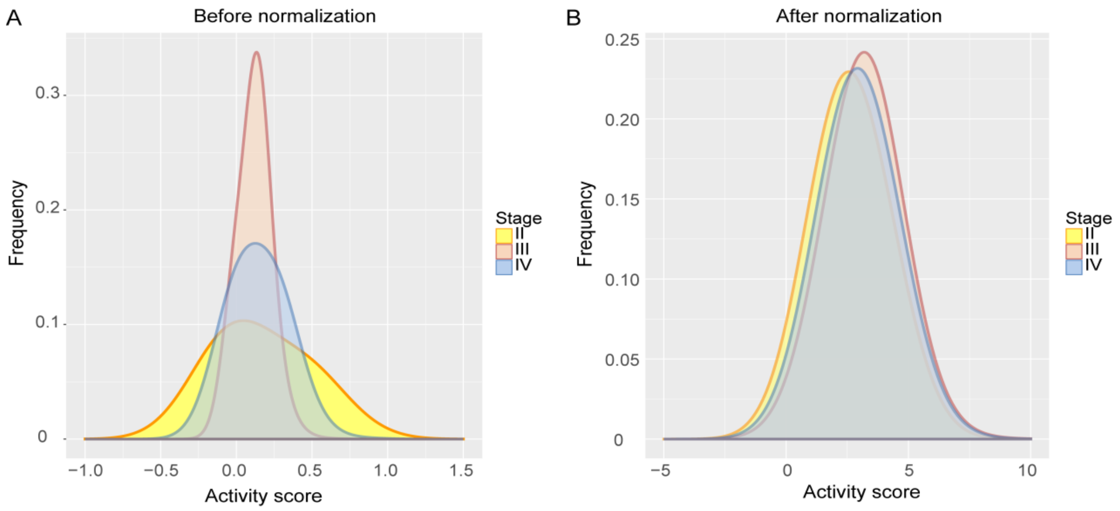

Figure S1. The distirbution of activity scores for TF-lncRNA regulations at different stages of OC. After normalization, the activity scores in each stage were required to have same data distribution.
