# Supplementary material for: Dynamic TF-lncRNA Regulatory Networks Revealed Prognostic Signatures in the Development of Ovarian Cancer
Source: Front Bioeng Biotechnol. 2020 May 13;8:460. doi: 10.3389/fbioe.2020.00460 (PMC7237576; doi:10.3389/fbioe.2020.00460)
Supplement: Supplementary file 10 [file Data_Sheet_2.PDF]

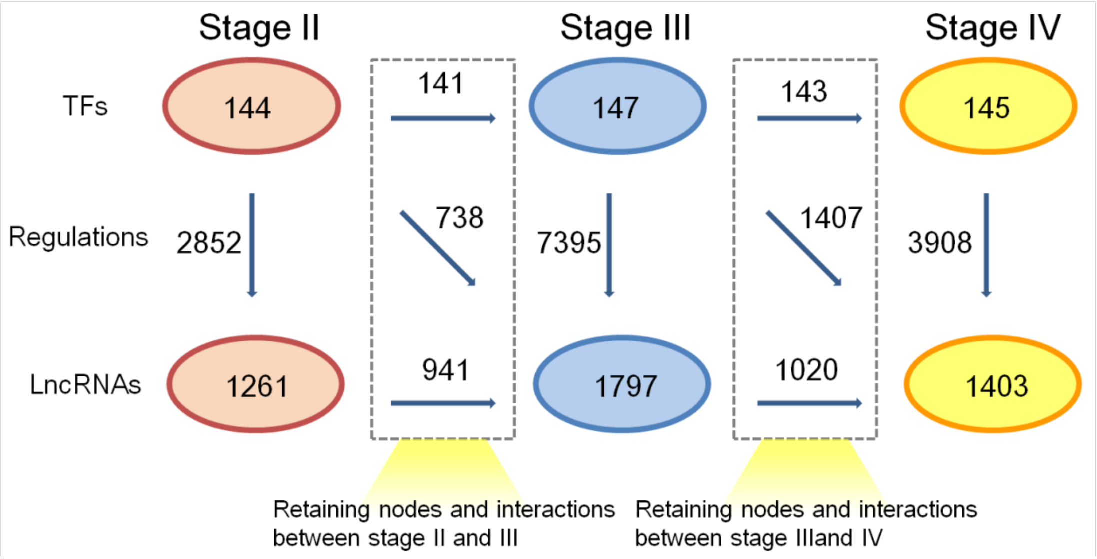

Figure S2. The dynamic retaining of TF-lncRNA nodes and regulations across different stages of OC.
