# Supplementary material for: Dynamic TF-lncRNA Regulatory Networks Revealed Prognostic Signatures in the Development of Ovarian Cancer
Source: Front Bioeng Biotechnol. 2020 May 13;8:460. doi: 10.3389/fbioe.2020.00460 (PMC7237576; doi:10.3389/fbioe.2020.00460)
Supplement: Supplementary file 11 [file Data_Sheet_3.PDF]

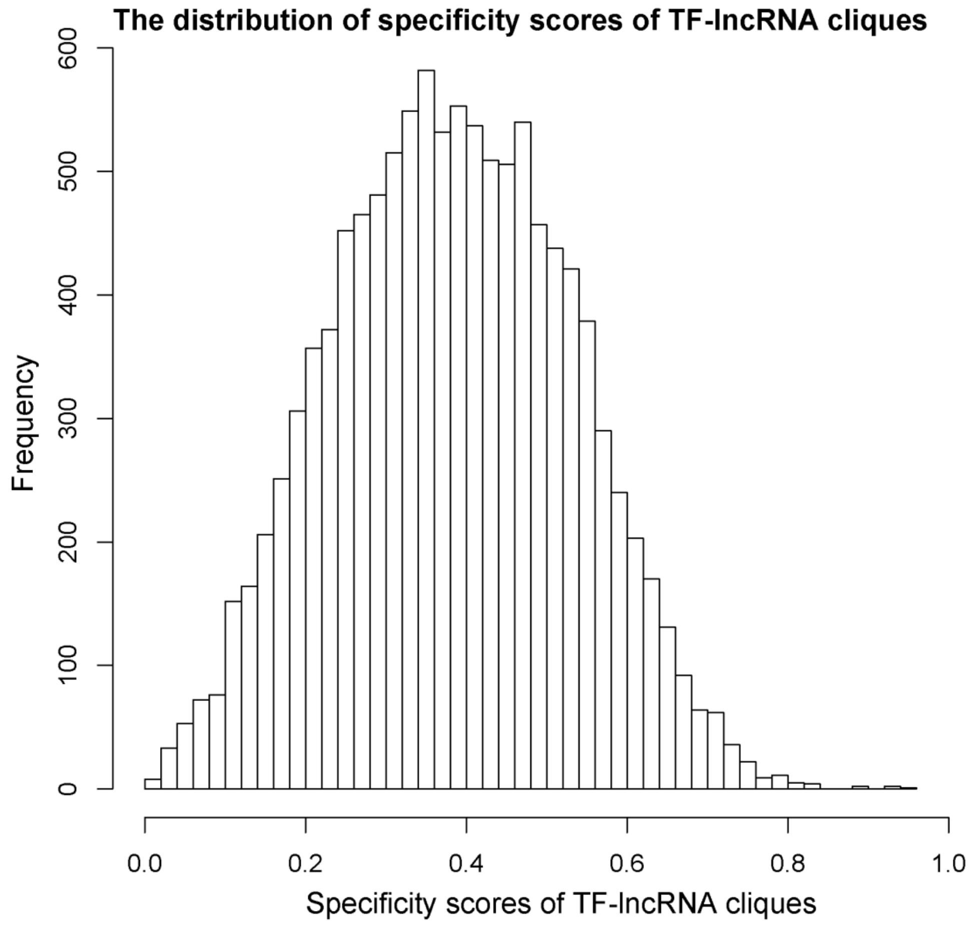

Figure S3. The distribution of specificity scores of TF-lncRNA regulatory cliques. More than 94% of these regulations have a specificity score > 0.15.
